# Chikungunya Virus Mosquito Vectors: A Global Review of Field Surveillance and Laboratory Vector Competence (2020–2026)

**DOI:** 10.3390/insects17080796

**Published:** 2026-07-31

**Authors:** Wei Yin Vinnie-Siow, Tiong Kai Tan, Sing Sin Sam, Yvonne Ai-Lian Lim, Boon Teong Teoh, Van Lun Low

**Affiliations:** 1 Higher Institution Center of Excellence, Tropical Infectious Diseases Research and Education Centre (TIDREC), Universiti Malaya, Kuala Lumpur 50603, Malaysia; vinnie_siow@um.edu.my (W.Y.V.-S.); singsin@um.edu.my (S.S.S.); boonteong@um.edu.my (B.T.T.); 2 Department of Parasitology, Faculty of Medicine, Universiti Malaya, Kuala Lumpur 50603, Malaysia; limailian@um.edu.my

**Keywords:** chikungunya virus, CHIKV, mosquito vectors, *Aedes aegypti*, *Aedes albopictus*, vector competence, field surveillance, ECSA, Indian Ocean lineage, integrated surveillance and control

## Abstract

The chikungunya virus (CHIKV) is an emerging alphavirus transmitted primarily by *Aedes* mosquitoes. This narrative review synthesised evidence from 56 mosquito-based records (34 field surveillance records and 22 laboratory vector competence records) published from January 2020 to June 2026. *Aedes aegypti* was the most frequently detected species, confirmed as CHIKV-positive in 22 of the 29 field studies that examined it, followed by *Aedes albopictus* (positive in nine of 12). Both species demonstrated consistent laboratory competence. Descriptive laboratory analysis showed mean infection, dissemination, and transmission values of approximately 76%, 77%, and 37% for *Ae. aegypti*, and 79%, 80%, and 47% for *Ae. albopictus*, respectively; these values are descriptive summaries across heterogeneous laboratory protocols and inconsistently reported genotypes. East/Central/South African–Indian Ocean Lineage of the chikungunya virus (ECSA-IOL) showed the highest mean transmission efficiency among genotype groups. In addition, CHIKV RNA was also detected in *Culex quinquefasciatus*, *Psorophora* spp., *Wyeomyia* spp., and other culicids in field studies, but laboratory evidence does not support these species as confirmed competent vectors. Other *Aedes* species, including *Aedes vittatus*, *Aedes polynesiensis*, *Aedes koreicus*, and *Aedes japonicus*, may have regional relevance but require further validation. Key evidence gaps include incomplete genotype reporting, limited virus isolation from positive mosquito pools, and inconsistent transmission metrics. Future preparedness for CHIKV emergence and spread requires integrated surveillance combining field detection, virus isolation, genomic sequencing, and standardised vector competence assays within an integrated surveillance framework.

## Simple Summary

The chikungunya virus (CHIKV) is an emerging alphavirus transmitted primarily by *Aedes* mosquitoes. This review synthesised evidence from 56 mosquito-based records (34 field surveillance records and 22 laboratory vector competence records) published from January 2020 to June 2026. *Aedes aegypti* was the most frequently detected species, confirmed as CHIKV-positive in 22 of the 29 field studies that examined it, followed by *Aedes albopictus* (positive in nine of 12).

## 1. Introduction

The chikungunya virus (CHIKV) is an alphavirus in the family Togaviridae and is an important cause of mosquito-borne febrile and arthritic disease. Human infection typically presents with acute fever, rash, headache, myalgia, and severe arthralgia. Although many cases resolve, persistent joint pain can last for months or even years, leading to long-term morbidity, reduced productivity, and substantial public health burden [1]. Over recent decades, CHIKV has triggered outbreaks across Africa, Asia, the Indian Ocean region, Europe, and the Americas, highlighting its capacity for geographic expansion and repeated re-emergence [2,3]. As of December 2024, local CHIKV transmission has been reported in 119 countries and territories [1], and global incidence rose from 0.28 to 11.13 per 100,000 population between 2004 and 2024, with the Americas bearing the highest burden [4].

Transmission dynamics are strongly influenced by mosquito ecology, especially the biology of *Aedes aegypti* (Linnaeus, 1762) and *Aedes albopictus* (Skuse, 1894) [1]. *Aedes aegypti* is highly adapted to urban settings, breeds readily in artificial containers, feeds on humans, and is closely linked to household-level transmission. These traits facilitate viral acquisition from viraemic hosts and rapid outbreak amplification in densely populated areas. On the other hand, *Ae. albopictus* exhibits greater ecological plasticity, thriving in all urban, peri-urban, rural, and vegetated environments. Its ongoing expansion into temperate regions has raised concerns about the potential for CHIKV establishment beyond historically tropical zones [5,6].

Multiple CHIKV genotypes and lineages have been identified, which include the Asian genotype, West African genotype, East/Central/South African genotype (ECSA), and the Indian Ocean lineage (IOL), which is derived from ECSA. These classifications are epidemiologically relevant, as the virus genotype may influence mosquito infection, dissemination, and transmission efficiency. ECSA and ECSA-IOL strains have been linked to major outbreaks and are frequently examined in laboratory vector competence studies [7,8,9].

A small number of adaptive mutations in the viral genome also shape CHIKV–vector interactions. The best characterised is the E1-A226V substitution in the E1 envelope glycoprotein, which arose independently within the Indian Ocean lineage (IOL) that emerged from the ECSA genotype. This single amino acid change enhances viral infectivity, replication, and dissemination in *Ae. albopictus*, shortening the extrinsic incubation period and improving transmission efficiency within the species; it is widely regarded as a key driver of the explosive IOL-associated outbreaks in the Indian Ocean region, India, and, subsequently, temperate regions where *Ae. albopictus* predominates [6,10,11]. Other substitutions, including the E2 mutations E2-L210Q and E2-K252Q, as well as additional E1 changes, can modulate midgut infection and dissemination, and may act epistatically with E1-A226V. These genotype-specific effects mean that a mosquito’s capacity to transmit CHIKV depends not just on the infecting virus strain but on the mosquito species as well.

Determining CHIKV vector status relies on both field and laboratory evidence. Field surveillance provides ecological evidence of mosquito-virus contact through the detection of CHIKV-positive specimens collected under natural conditions. Laboratory studies, in turn, assess whether mosquitoes can acquire the virus, support its dissemination, and transmit it via saliva. However, field RNA detection alone is insufficient to confirm vector competence, as it may reflect a recent infectious blood meal, non-infectious virus, abortive infection (in which the virus enters midgut cells but fails to establish a productive, disseminating infection), or contamination. Moreover, mosquito infection rate estimates are sensitive to sampling design, pool size, and detection probability. Thus, vector status should be assigned based on a combination of field detection, virus isolation, genomic confirmation, and laboratory evidence of transmission [12,13,14,15,16].

This review synthesises studies published between January 2020 and June 2026 on mosquito vectors of CHIKV, with an emphasis on field surveillance, laboratory vector competence, viral genotypes, country-level patterns, temporal trends, infection metrics, diagnostic approaches, and integrated surveillance considerations. The objective is to distinguish confirmed vectors from potential regional vectors and identify field-positive species that require further experimental validation.

## 2. Literature Search and Data Extraction

This is a narrative review; the structured search described below was undertaken to ensure transparency and reproducibility rather than to constitute a systematic or scoping review, and accordingly, no PRISMA flow diagram or formal risk-of-bias assessment was performed. A structured literature search was conducted using PubMed and Google Scholar to identify relevant studies published from 1 January 2020 to 18 June 2026. Two search strategies were applied to capture the two major evidence categories included in this review. The first search focused on field surveillance studies reporting CHIKV detection in field-collected mosquitoes, while the second search focused on laboratory vector competence studies evaluating experimental CHIKV infection and transmission in mosquitoes.

The literature search was designed to identify original mosquito-based CHIKV studies, including both field surveillance and laboratory vector competence investigations. Search terms combined CHIKV-related keywords with mosquito, surveillance, and vector competence terms, including “chikungunya”, “chikungunya virus”, “CHIKV”, “mosquito”, “*Aedes*”, “*Culex*”, “*Anopheles*”, “field-caught”, “field-collected”, “entomological surveillance”, “mosquito surveillance”, “arbovirus surveillance”, “minimum infection rate”, “MIR”, “virus isolation”, “RT-PCR”, “vector competence”, “oral infection”, “experimental infection”, “blood meal”, “extrinsic incubation”, “saliva”, “infection rate”, “dissemination rate”, and “transmission efficiency”.

Retrieved articles were screened first by title and abstract, followed by full-text assessment. Studies were included if they reported original data on CHIKV detection in field-collected mosquitoes or laboratory evaluations of mosquito vector competence for CHIKV. Reviews, systematic reviews, meta-analyses, case reports, vaccine-focused studies, human clinical studies without mosquito data, and studies lacking relevant entomological outcomes were excluded.

Countries are represented according to the published, indexed mosquito-based CHIKV studies that met the inclusion criteria rather than through selective inclusion. Duplicate records identified across both databases were removed manually, while the grey literature and non-indexed sources were not systematically searched.

Data were extracted into two standardised evidence tables: one for field surveillance studies and one for laboratory vector competence studies. Extracted variables included the authors, year of publication, country or mosquito origin, mosquito species, CHIKV strain or genotype, CHIKV strain group, sample size, infection rate (IR), minimum infection rate (MIR), dissemination rate (DR), transmission rate/transmission efficiency (TR/TE), extrinsic incubation period, incubation temperature, and detection method. Where applicable, CHIKV strains were grouped as Asian, West African, East/Central/South African (ECSA), ECSA–Indian Ocean lineage (ECSA-IOL), mixed Asian/ECSA-IOL, or not reported. Descriptive counts and means were calculated directly from the latest field and laboratory tables. Infection rate (IR) and dissemination rate (DR) were summarised using row-level mean values when multiple time points or populations were reported, while transmission efficiency/transmission rate (TE/TR) used the maximum reported value when no overall transmission estimate was available, which was chosen to capture the upper bound of demonstrated transmission potential rather than to represent a typical rate. These values should be interpreted as descriptive summaries rather than pooled estimates because mosquito infection rate and minimum infection rate (MIR) estimates can be biassed by pool size, sampling intensity, and detection probability.

All numerical comparisons in this manuscript are descriptive. Field-positive and MIR values were not pooled statistically because the studies differed in sampling design, pool size, outbreak timing, and detection method; laboratory rates were also summarised descriptively, as experiments differed in mosquito population, virus strain, infectious dose, incubation temperature, extrinsic incubation period, and saliva assay method.

## 3. Overall Summary of Mosquito-Based CHIKV Records

The review included 56 mosquito-based records from the latest dataset: 34 field surveillance records and 22 laboratory vector competence records. Field surveillance records documented CHIKV detection in naturally collected mosquitoes, whereas laboratory records evaluated experimental infection, dissemination, or transmission (Table 1 and Table 2). Of the 34 field surveillance records, 28 yielded confirmed CHIKV detection in at least one mosquito species, while the remaining six screened mosquitoes without detecting CHIKV (Table 1).

**Figure 1:**
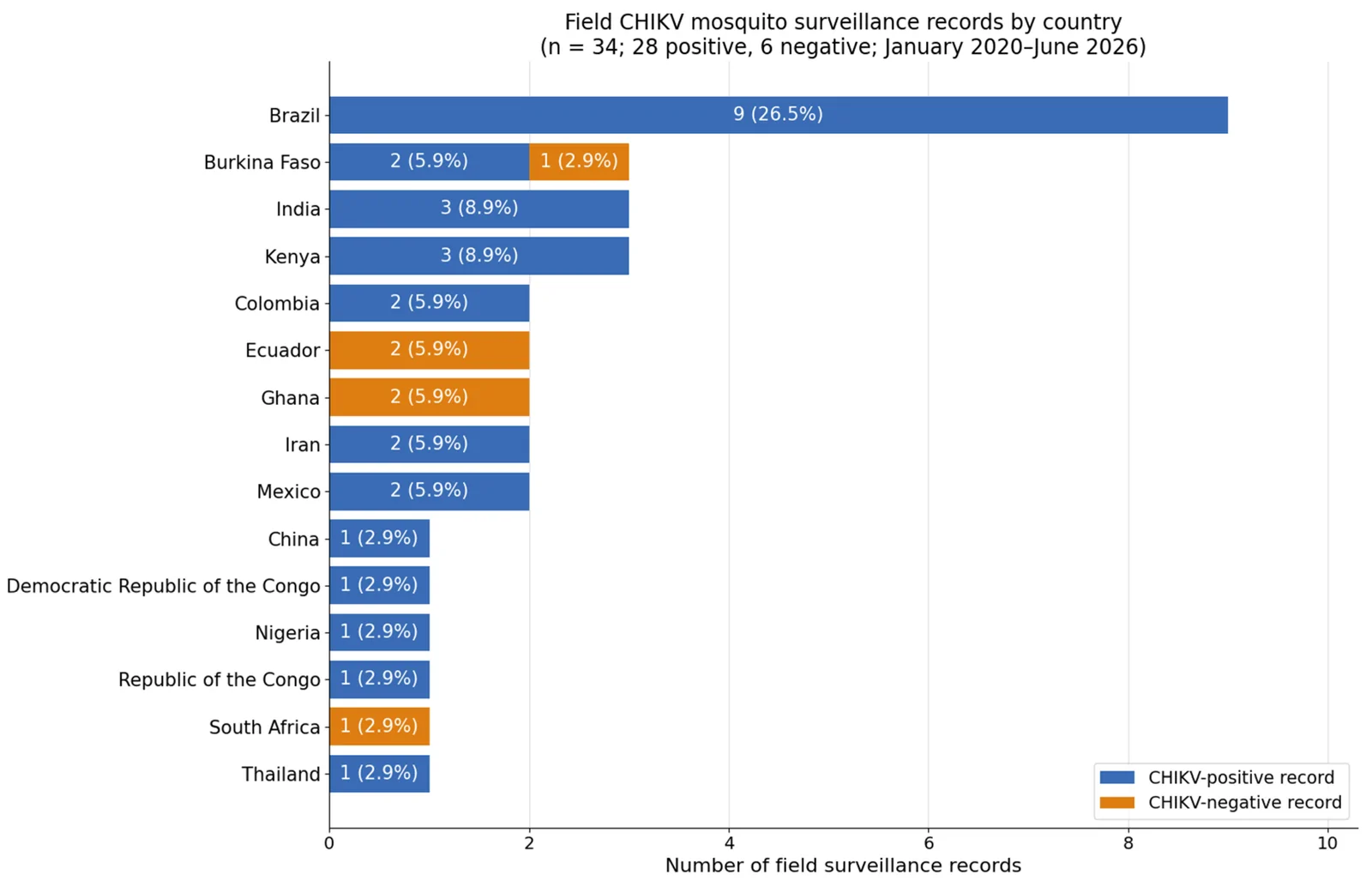
Country distribution of field CHIKV mosquito surveillance records (*n* = 34; 28 with confirmed CHIKV detection and six as CHIKV-negative), January 2020–June 2026. Bars are stacked to show CHIKV-positive (blue) and CHIKV-negative (orange) records for each country.

The field records were distributed across 15 countries or territories. Brazil contributed the highest number of records (9/34; 26.5%), followed by India, Kenya, and Burkina Faso (three records each). Ecuador, Iran, Colombia, Mexico, and Ghana contributed two records each, while the Republic of the Congo, the Democratic Republic of the Congo, Thailand, South Africa, China, and Nigeria each contributed one record (Table 1 and Figure 1). Surveillance in Ecuador, Ghana, and South Africa (five records in total) detected no CHIKV in any of the species screened, and one of the three Burkina Faso records was likewise negative; the remaining 28 field records each confirmed CHIKV in at least one mosquito species.

The field dataset contained a broader range of mosquito species than the laboratory dataset because field studies often collected mixed mosquito communities during outbreaks, endemic or ecological surveillance, and screened pools for viral RNA (Table 1). On the other hand, laboratory studies more commonly focused on known or suspected vector species and measured biological outcomes, such as infection, dissemination, and saliva-based transmission (Table 2).

### 3.1. Yearly Trend of Mosquito-Based CHIKV Records

**Figure 2:**
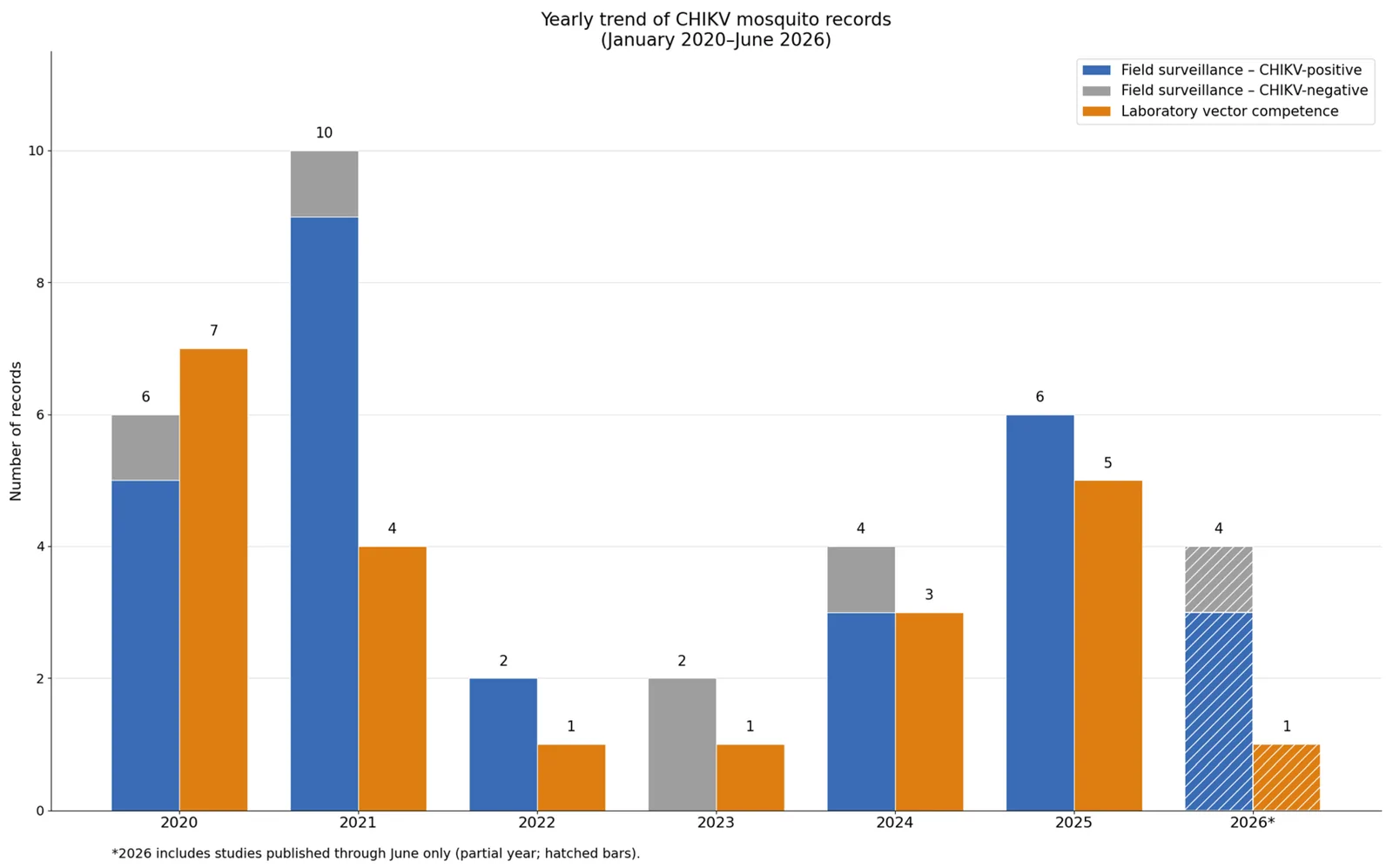
Yearly trend of field surveillance and laboratory vector competence records included in this review, January 2020–June 2026. Field-surveillance bars are stacked to show CHIKV-positive (blue) and CHIKV-negative (grey) records, and laboratory vector competence records (orange) are shown alongside for each year. Bars for 2026 (asterisk, hatched) include only the studies published up to June.

The yearly distribution of records showed that field surveillance and laboratory vector competence studies varied across the review period (Figure 2). Field surveillance records peaked in 2021 with 10 records, followed by 2025 with six records and 2020 with six records. Laboratory vector competence records were highest in 2020 with seven records, followed by 2025 with five records. Both evidence types were least represented in 2022 and 2023 (two field surveillance records and one laboratory record in each year).

Overall, there were more field surveillance studies than laboratory vector competence studies across most years, reflecting the larger contribution of mosquito screening studies to the dataset. The increase in both field and laboratory records in 2024 and 2025 suggests growing research attention to CHIKV mosquito surveillance and vector competence. However, the lower number of records in 2026 should be interpreted cautiously, as the review only included studies published up to June 2026.

### 3.2. Geographical Summary of CHIKV Mosquito Field Surveillance Records

#### 3.2.1. The Americas

Field surveillance in the Americas was dominated by *Aedes aegypti* and concentrated in Brazil, which contributed the most records of any country (nine of 34; 26.5%) across several states, including Pará, Rio Grande do Norte, Bahia, Mato Grosso, Paraná, Minas Gerais, São Paulo, and Goiás. All nine Brazilian records were CHIKV-positive for *Ae. aegypti*, while several Brazilian studies also reported viral RNA in *Ae. albopictus*; *Culex quinquefasciatus* (Say, 1823); *Aedes fluviatilis* (Lutz, 1904); *Wyeomyia bourrouli* (Lutz, 1905); *Psorophora ferox* (Humboldt, 1819); *Psorophora albigenu* (Peryassú, 1908); and other culicids, and a dedicated review of CHIKV’s spatiotemporal distribution and genotype patterns in Brazil highlighted *Ae. albopictus* as a potential secondary vector in regions where the E1-A226V mutation is present [11]. Where genotype was reported, most Brazilian detections involved ECSA strains [17,18,19,20] while one record was classified as ECSA-IOL [21], with the data indicating sustained CHIKV circulation between 2020 and 2025.

The repeated detection of CHIKV in *Ae. aegypti* is consistent with its role in urban transmission, whereas its detection in *Ae. albopictus* suggests possible involvement in peri-urban or mixed ecological settings. Reports involving *Culex*, *Psorophora*, and *Wyeomyia* mosquitoes can only indicate that CHIKV RNA was present in field-collected specimens and cannot establish these species as competent vectors; *Cx. quinquefasciatus* has been examined in only two laboratory vector competence studies (Table 2), with low and inconsistent outcomes, while *Psorophora* and *Wyeomyia* have not been experimentally evaluated, so these detections are best read as ecological exposure rather than competence. The same pattern recurred elsewhere in the Americas. In Ecuador, surveillance focused on human-biting *Ae. aegypti* in both Quinindé [22] and in Santo Domingo de los Tsáchilas, with the latter also including some *Ae. albopictus* [23], but no CHIKV was detected in either study. In Colombia, CHIKV was detected in *Ae. aegypti* in Ibagué [24] and in both *Ae. aegypti* and *Ae. albopictus* in Cauca [25], indicating that both species may contribute to transmission and warrant surveillance. In Mexico, surveillance targeted *Ae. aegypti* exclusively, with natural infection of indoor females in Mérida [26] and an Asian-genotype detected in Ciudad Juárez [27]. Across the Americas, *Ae. aegypti* was consistently the principal field-detected vector, while *Ae. albopictus* emerged as a secondary peri-urban species, and non-*Aedes* detections reflected exposure rather than confirmed transmission.

#### 3.2.2. Africa

African surveillance spanned East, West, and Central Africa, and repeatedly recovered *Ae. aegypti* alongside a wider range of *Aedes* and non-*Aedes* species. In Kenya, three studies involved *Ae. aegypti*, *Aedes vittatus* (Bigot, 1861), and *Cx. quinquefasciatus*, with two records classified as ECSA [15,28] and one genotype not reported [29]; CHIKV detection in *Ae. aegypti* is consistent with its role as a major vector, whereas RNA detection in *Ae. vittatus* and *Cx. quinquefasciatus*, as is the case for other species besides *Ae. aegypti*, documents outbreak-period exposure but does not establish competence by itself, which requires experimental confirmation (Section 4). In Burkina Faso, three studies conducted in Bobo-Dioulasso and Pouytenga mainly involved *Ae. aegypti*, wherein Gomgnimbou et al. sampled *Ae. aegypti*, *Aedes furcifer* (Edwards, 1913), and *Ae. vittatus* but detected no CHIKV [30]; meanwhile, Laouali et al. reported ECSA-classified CHIKV in *Ae. aegypti* pools from Bobo-Dioulasso [31], and Toé et al. reported its circulation in *Ae. aegypti* during the 2023 Pouytenga outbreak [32], together indicating a central role for *Ae. aegypti* in outbreak-related surveillance.

In Central Africa, single records from the Republic of the Congo (*Ae. aegypti*, *Ae. albopictus*, and *Culex* spp. [33]) and from Matadi in the Democratic Republic of the Congo (*Ae. albopictus* in an outbreak setting [34]) indicated ECSA-related circulation; of the species collected, CHIKV was confirmed in *Ae. albopictus* (a positive pool in the Republic of the Congo, and detections during the Matadi outbreak), whereas *Ae. aegypti* and *Culex* spp.’s pools tested negative (Table 1). West and South African surveillance added further breadth: Ghanaian studies contributed *Aedes*-focused arbovirus surveillance without detecting CHIKV [35,36]; a South African study screened diverse *Aedes*, *Culex*, *Anopheles*, and *Mansonia* species but detected no CHIKV [37]; and nationwide surveillance in Nigeria detected CHIKV in multiple *Aedes* species, including *Ae. aegypti* and *Aedes luteocephalus* (Newstead, 1907) [38]. Across Africa, *Ae. aegypti* again dominated the field evidence, while the recurrent detection of *Ae. vittatus* and other forest-associated or non-*Aedes* species underscores the ecological complexity of African settings and the need for experimental confirmation of any secondary vector roles.

#### 3.2.3. Asia

Asian surveillance combined routine xenomonitoring with newer molecular approaches. India contributed three 2021 records that used molecular xenomonitoring of CHIKV and other arboviruses in *Ae. aegypti* and *Ae. albopictus*, with one ECSA-classified record [39] and two without genotypes [40,41], highlighting the value of integrated arbovirus surveillance where dengue, chikungunya, and Zika co-circulate in shared vector populations. Iran contributed two contrasting records: Bakhshi et al. detected the Asian-genotype CHIKV RNA in *Culiseta longiareolata* (Macquart, 1838), *Culex tritaeniorhynchus* (Giles, 1901), and *Anopheles maculipennis* (Meigen, 1818) s.l. [42]—as these are not classical CHIKV vectors, this should be interpreted as exposure rather than competence—while Abbasi et al. used metagenomic sequencing across *Cx. quinquefasciatus*; *Culex pipiens* Linnaeus, 1758; *Ae. aegypti*; *Ae. albopictus*; *Anopheles stephensi* (Liston, 1901); and *Anopheles culicifacies* (Giles, 1901), classifying the virus as ECSA with positivity in *Ae. albopictus* and *Ae. aegypti* [43]—here, confirmed positivity in the recognised *Aedes* vectors indicates active circulation, whereas *Culex* and *Anopheles* detections reflect exposure requiring experimental validation. Thailand contributed a single but instructive record in which CHIKV RNA was detected in field-collected *Cx. quinquefasciatus* from Bangkok; however, laboratory follow-up did not support productive replication, illustrating the value of distinguishing field detection from vector competence [16]. Finally, China reported whole-genome CHIKV detection during the Foshan outbreak (ECSA-IOL [44]); whole-genome sequencing from field-collected mosquitoes confirms an intact viral genome, enables accurate genotype assignment, and provides stronger molecular evidence of field infection than partial RNA detection alone. Together, the Asian records illustrate the growing application of whole-genome and metagenomic sequencing during 2020–2026; while RT-PCR and qPCR have been the mainstay of arbovirus detection since the early 2000s, many field studies still relied on molecular detection without sequence confirmation, which contributed to incomplete genotype reporting (Table 1).

#### 3.2.4. Mediterranean and Europe

The search returned no indexed 2020–2026 field surveillance records reporting CHIKV detection in mosquitoes from mainland Europe. The European and Mediterranean evidence is therefore represented in this review through laboratory vector competence studies rather than field collections [9,45,46,47]. This gap most likely reflects publication timing rather than an absence of risk, and targeted European entomological surveillance is flagged as a priority.

**Table 1:** Published field surveillance studies reporting CHIKV detection in mosquitoes, 2020–2026.

No.	Authors	Year	Country/Area	Locality	Mosquito Species	CHIKV Genotype	Sample Size	Infection Rate/ Positive Pools (%)	MIR (%)	Detection Method
1	Bakhshi et al. [42]	2020	Iran	Iran (North Khorasan, Mazandaran, and Fars provinces)	*Cs. longiareolata*; *Cx. tritaeniorhynchus*; *An. maculipennis* s.l.	Asian	*Cs. longiareolata*, 34; *Cx. tritaeniorhynchus*, 129; *An. maculipennis* s.l., 514	*Cs. longiareolata*: 5.9% (2/34); *Cx. tritaeniorhynchus*: 1.6% (2/129); *An. maculipennis* s.l.: 0.4% (2/514)	NR	RT-PCR/qPCR
2	Cruz et al. [17]	2020	Brazil	Xinguara, Pará, Brazil	*Ae. aegypti*; *Cx. quinquefasciatus*	ECSA	492 (36 pools)	*Ae. aegypti*: 19.4% (7/36 pools); *Cx. quinquefasciatus*: 2.8% (1/36 pools)	NR	RT-PCR/qPCR; IFA; virus isolation
3	De Melo Ximenes et al. [21]	2020	Brazil	Natal, Rio Grande do Norte, Brazil	*Ae. aegypti*; *Ae. albopictus* *Ae. fluviatilis*; *Wyeomyia bourrouli*; *Hg. leucocelaenus*; *Ae. serratus*; *Ae. taeniorhynchus*; *Ae. scapularis*	ECSA-IOL	Total: 314 mosquitoes *Wy. bourrouli*: 210, *Ae. albopictus*: 73, *Ae. fluviatilis*: 10, *Hg. leucocelaenus*: 7, *Ae. scapularis*: 7, *Ae. aegypti*: 5, *Ae. serratus*: 1, *Ae. taeniorhynchus*: 1	CHIKV-positive species detected; infection rate by species not reported (*Ae. aegypti*, *Ae. albopictus*, *Ae. fluviatilis*, *Wy. bourrouli*)	NR	RT-PCR
4	Vairo et al. [33]	2020	Republic of the Congo	Republic of the Congo (Kouilou and Pointe-Noire)	*Ae. aegypti*; *Ae. albopictus*; *Culex.* spp.	ECSA-IOL	*Ae. albopictus*: Nkoungou, 18; Diosso, 61; Mengo, 8; Matombi, 16; Mabindou, 25, Mpita, 34; Tchiamba Nzassi, 47; Siafoumou, 10 *Ae. aegypti*: Mptiya, 20; Diosso, 2 Culicinae spp.: 12	*Ae. albopictus*: 1 positive pool; *Ae. aegypti*: negative; Culicinae spp.: negative	NR	RT-PCR/qPCR
5	Ortega-López et al. [22]	2020	Ecuador	Quinindé, Ecuador	*Ae. aegypti*	NR	429 individuals	Negative	Negative	RT-PCR
6	Heath et al. [29]	2020	Kenya	Kenya (Kisumu, Chulaimbo, Ukunda, Msambweni)	*Ae. aegypti*	NR	714 pools	5.9% (43/714 pools)	0.49% (0.34–0.68%)	RT-PCR/qPCR
7	Teixeira et al. [18]	2021	Brazil	Vitória da Conquista, Bahia State, Brazil	*Ae. aegypti*	ECSA	480 (450 larvae in 30 pools of 25, plus 30 individual larvae)	13.3% (4/30 pools)	4.3%	RT-PCR/qPCR
8	Chand et al. [39]	2021	India	Jabalpur and Narsinghpur districts, Central India	*Ae. aegypti*; *Ae. albopictus*; *Ae. vittatus*	ECSA	Total mosquitoes: 2991 *Ae. aegypti*: 2831, *Ae. albopictus*: 149, *Ae. vittatus*: 11	*Ae. aegypti*: 1.27% (5/394 pools)	*Ae. aegypti*: 0.36%	RT-PCR/qPCR; plaque assay
9	Lutomiah et al. [15]	2021	Kenya	Mombasa, Kenya	*Ae. aegypti*; *Ae. vittatus*; *Cx. quinquefasciatus*	ECSA	Total mosquitoes: 6899 adults; *Ae. aegypti*: 911; *Ae. vittatus*: 1137; *Cx. quinquefasciatus*: 4492	*Ae. aegypti*: 0.87% (1/115 pools); *Ae. vittatus*: negative; *Cx. quinquefasciatus*: 1.65% (4/243 pools)	*Ae. aegypti*(F): 0.30%; *Cx. quinquefasciatus* (F): 0.08%; *Cx. quinquefasciatus* (M): 0.10%	RT-PCR/qPCR; virus isolation
10	Weggheleire et al. [34]	2021	Democratic Republic of the Congo	Matadi, Democratic Republic of the Congo (DRC)	*Ae. aegypti*; *Ae. albopictus*	ECSA	11 adult mosquito pools; 15 larvae pools	*Ae. albopictus*: 45.5% (5/11 adult pools); 13.3% (2/15 larval pools)	0–1.31%	RT-PCR/qPCR; ELISA
11	Phumee et al. [16]	2021	Thailand	Bangkok, Thailand	*Cx. quinquefasciatus*	ECSA-IOL	43 (16 males, 27 females)	18.6% (8/43 mosquitoes)	NR	RT-PCR/qPCR; IFA; virus isolation
12	Da Silva-Neves et al. [48]	2022	Brazil	Cuiabá and Várzea Grande, Mato Grosso, Brazil	*Ae. aegypti*; *Ae. albopictus*; *Cx. quinquefasciatus*; *Ps. albigenu*; *Ps. ferox*	NR	5040 adults (2873 males; 2167 females) in 398 pools	*Ae. aegypti* F: 6.52% (3/46); *Ae. aegypti* M: 6.06% (2/33); *Ae. albopictus* F: 13.79% (4/29); *Cx. quinquefasciatus* F: 21.35% (19/89); *Cx. quinquefasciatus* M: 13.13% (13/99); *Ps. albigenu* F: 16.67% (2/12); *Ps. ferox* F: 12.50% (2/16)	NR	RT-PCR/qPCR
13	Carrasquilla et al. [24]	2021	Colombia	Ibagué, Colombia	*Ae. aegypti*	NR	*Ae. aegypti*: 482 individuals (133 pools), 317 individuals; *Ae. albopictus*: 7	*Ae. aegypti*: 0.8%	0.21%	RT-PCR/qPCR
14	Joannides et al. [35]	2021	Ghana	Mole Game Reserve and Larabanga, Northern Ghana	*Ae. aegypti*; *Ae. vittatus*	NR	1957 (75 pools)	Negative	NA	RT-PCR/qPCR
15	Munivenkatappa et al. [40]	2021	India	Karnataka State, India	*Ae. aegypti*	NR	50 pools	10% (5/50 pools)	NR	PCR
16	Vikram et al. [41]	2021	India	Delhi, India	*Ae. aegypti*	NR	981 individuals	13.0%	NR	RT-PCR/qPCR
17	Kirstein et al. [26]	2021	Mexico	Mérida, Yucatán, Mexico	*Ae. aegypti*	NR	2161 individuals	6.0% (129/2161 females)	NR	RT-PCR/qPCR
18	Leandro et al. [49]	2022	Brazil	Foz do Iguaçu, Brazil	*Ae. aegypti*	NR	109 individuals	5.45% (3/55)	NR	RT-PCR/qPCR; plaque assay
19	Akyea-Bobi et al. [36]	2023	Ghana	Accra, Ghana (Madina, Achimota Forest)	*Ae. aegypti*; *Ae. albopictus*	NR	*Ae. aegypti*: 1493 (120 pools); *Ae. albopictus*: 1 pool	Negative	NA	RT-PCR/qPCR
20	Guarido et al. [37]	2023	South Africa	South Africa (Gauteng, Limpopo, Northwest, Mpumalanga, KwaZulu-Natal)	*Ae. durbanensis*; *Ae. mcintoshi*; *Cx. pipiens* s.l.; *Cx. univittatus*; *Anopheles* spp.; *Mansonia* spp.	NR	39,035 (1462 pools)	Negative	NA	RT-PCR/qPCR
21	Almeida-Souza et al. [19]	2024	Brazil	Salinas, Minas Gerais, Brazil	*Ae. aegypti*; *Cx. quinquefasciatus*	ECSA	Total: 421 individuals *Ae. aegypti*: 31 pools; *Cx. quinquefasciatus*: 26 pools	*Ae. aegypti*: 32.3% (10/31); *Cx. quinquefasciatus*: 7.7% (2/26)	*Ae. aegypti*: 6.06%; *Cx. quinquefasciatus*: 0.78%	RT-PCR/qPCR
22	Banho et al. [20]	2024	Brazil	São José do Rio Preto, São Paulo, Brazil	*Ae. aegypti*; *Ae. albopictus*; *Aedes* spp.; *Culex* spp.	ECSA	Total: 1183 *Ae. aegypti*: 744; *Ae. albopictus*: 11; *Aedes* sp.: 1; *Culex* spp.: 427	*Ae. aegypti* females: 33% (26/79); *Ae. aegypti* males: 34% (27/79); *Ae. albopictus* females: 2.5% (2/79); *Culex* spp. females: 21.5% (17/79); *Culex* spp. males: 8.8% (7/79)	NR	RT-PCR/qPCR
23	Silva et al. [50]	2024	Brazil	Goiânia, Goiás, Brazil	* Ae. aegypti *	NR	1570 (157 pools)	1.3% (2/157 pools)	0.127%	RT-PCR/qPCR
24	Gomgnimbou et al. [30]	2024	Burkina Faso	Bobo-Dioulasso, Burkina Faso	*Ae. aegypti*; *Ae. furcifer*; *Ae. vittatus*	NR	Total: 976 individuals *Ae. aegypti*: 959; *Ae. furcifer*: 6; *Ae. vittatus*: 11	Negative	NA	RT-PCR/qPCR
25	Hernández-Acosta et al. [27]	2025	Mexico	Ciudad Juárez, Chihuahua, Mexico	*Ae. aegypti*	Asian	328 individuals	65.65%	26.0%	RT-PCR/qPCR
26	Banho et al. [51]	2025	Brazil	São José do Rio Preto, São Paulo, Brazil	*Ae. aegypti*; *Ae. albopictus*; *Culex* spp.	ECSA	1914 individuals	Total: 5.6% (107/1914) *Ae. aegypti* F: 32.7% (35/107); *Ae. aegypti* M: 28.0% (30/107); *Ae. albopictus * F: 1.9% (2/107; *Culex* spp. F: 26.2% (28/107); *Culex* spp. M: 11.2% (12/107)	NR	RT-PCR/qPCR; virus isolation
27	Musili et al. [28]	2025	Kenya	Mombasa, Kenya	*Ae. vittatus*	ECSA	2989 (842 pools)	0.12% (1/842)	NR	Virus isolation/NGS
28	Zhou et al. [44]	2025	China	Foshan City, Guangdong, China	*Ae. albopictus*	ECSA-IOL	1569 female	9.09% (7/77)	0.446% (up to 0.917% in Lecong Town)	RT-PCR/qPCR
29	Mantilla-Granados J.S. et al. [25]	2025	Colombia	Cauca, Colombia (Piamonte, Patía, Piendamó, Popayán)	*Ae. aegypti*; *Ae. albopictus*	NR	*Ae. aegypti*: 89 pools; *Ae. albopictus*: 34 females	*Ae. aegypti*: 12.4% (11/89); *Ae. albopictus*: 41.2% (14/34)	*Ae. aegypti*: 4.94%	RT-PCR/qPCR
30	Nwangwu et al. [38]	2025	Nigeria	Nigeria	*Ae. aegypti*; *Ae. luteocephalus*	NR	2406 (127 pools)	*Ae. aegypti*: 14.5% (9/62 pools); *Ae. luteocephalus*: 33.3% (1/3 pools)	*Ae. aegypti*: 0.16%; *Ae. luteocephalus*: 6.25%	RT-PCR/qPCR
31	Laouali et al. [31]	2026	Burkina Faso	Bobo-Dioulasso, Burkina Faso	*Ae. aegypti*	ECSA	23,229 individuals (199 pools)	1.0% (2/199 pools)	0.017%	RT-PCR/qPCR
32	Abbasi [43]	2026	Iran	Iran (Hormozgan, Sistan and Baluchestan, and Khuzestan provinces)	*Cx. quinquefasciatus*; *Ae. aegypti*; *Cx. pipiens*; *Ae. albopictus*; *An. stephensi*; *An. culicifacies*	ECSA	Total: 4275 individuals (152 pools, ranging from 10 to 30 individuals)	*Ae. albopictus*/ *Ae. aegypti*: 11.1% (infection rate specified by species)	NR	NGS/ sequencing
33	Toé et al. [32]	2026	Burkina Faso	Pouytenga, Burkina Faso	*Ae. aegypti*	NR	Total: 41(3 pools)	66.67% (2/3 pools)	4.88%	RT-PCR/qPCR
34	Herrera et al. [23]	2026	Ecuador	Santo Domingo de los Tsáchilas, Ecuador	*Ae. aegypti*; *Ae. albopictus*	NR	3918 (196 pools)	Negative	Negative	RT-PCR/qPCR

NR = not reported; NA = not applicable. Sample sizes are reported as given in the source studies; some give the number of individual mosquitoes tested, others the number of pools, and some both—where pools were screened, the pool count is shown in parentheses. Genus abbreviations: *Ae.* = *Aedes*; *An.* = *Anopheles*; *Cs.* = *Culiseta*; *Cx.* = *Culex*; *Ps.* = *Psorophora*; *Wy.* = *Wyeomyia*.

**Table 2:** Published laboratory vector competence studies of CHIKV in mosquitoes, 2020–2026.

No.	Authors	Year	Mosquito Species	Mosquito Origin	Strain Type	CHIKV Genotype	Sample Size	IR (%)	DR (%)	TE/TR (%)	EIP	Temp.	Detection Method
1	Robison et al. [52]	2020	*Ae. aegypti*	Poza Rica, Mexico	Lab	Asian	60 mosquitoes per time point from 2 to 18 dpi; 50 at 20 dpi	Midgut infection: 98% (59/60) at 2 dpi; 100% from 4 to 18 dpi; 94% (47/50) at 20 dpi	Legs/wings: 100% at 2–6, 10, 12, 16 dpi; 98% at 8, 14, 18 dpi; 96% at 20 dpi	Infectious saliva by plaque assay: 28% at 2 dpi, 27% at 4 dpi, 18% at 6 dpi, 10% at 8 dpi, 5% at 10 dpi, 0% at 12–18 dpi, 0% at 20 dpi	2 dpi	28 °C	RT-qPCR; plaque assay on Vero cells for infectious saliva
2	Bohers et al. [7]	2020	*Ae. albopictus*	Tunisia (Carthage/ Tunis)	Both	ECSA-IOL	134 individuals	76.7% at 3 dpi to 79.2% at 21 dpi Overall: 81.3% (109/134)	82.6% at 3 dpi to 94.7% at 21 dpi; Overall: 90.8%	TR 75% at 10 dpi; TE peaked at 55.5% at 10 dpi	3 dpi	28 ±1 °C	Focus fluorescent assay on C6/36 cells
3	Calvez et al. [53]	2020	*Ae. polynesiensis*	Wallis and Futuna Islands (WKANA: Kanahe; WLALO: Lalolalo lake area)	Field	Asian	WKANA: 31; WLALO: 26	WKANA: 83.9% (26/31); WLALO: 84.6% (22/26)	WKANA: 76.9% (20/26); WLALO: 77.3% (17/22)	WKANA: 45.0% (9/20); WLALO: 47.1% (8/17)	7 dpi	28 °C	RT-PCR/qPCR and infectious saliva/CPE assay
4	Shin et al. [54]	2020	*Ae. aegypti*	Vero Beach and Key West, Florida, USA	Both	ECSA-IOL	Total: 900 individuals; Subsets: 15 at 4 dpi and 43–75 at 10 dpi per population	4 dpi body: Rockefeller 73.3% (11/15); Vero Beach 93.3% (14/15); Key West 86.6% (13/15); 10 dpi body: Rockefeller 88.4% (38/43); Vero Beach 98.5% (64/65); Key West 97.3% (73/75)	10 dpi legs: Rockefeller 73.7% (28/38); Vero Beach 100% (64/64); Key West 86.3% (63/73)	NA	NR	28 °C	RT-qPCR/qPCR
5	Gutiérrez-Bugallo et al. [55]	2020	*Ae. aegypti*	Havana, Cuba (Pasteur/PTE, Parraga/PRG); Mombasa, Kenya	Field	ECSA	60 females per population per viral strain	95–100% at 3 dpe for both PTE and PRG	PTE 86%, PRG 100% at 14 dpe	PTE 4–14%; PRG 0–11%	7 dpi	28 °C	Plaque assay/infectious virus titration
6	Kaczmarek et al. [56]	2020	*Ae. albopictus*	New York City, USA	Both	ECSA-IOL	16/21/7 by population	>72% at 14 dpi	>72% at 14 dpi	3–10% at 7 dpi; 15% at 14 dpi; in vivo Tallman model: 60–80%	2 dpi	28 °C	Plaque assay; RT-qPCR; in vivo mouse transmission model; sequencing
7	Gloria-Soria et al. [57]	2020	*Ae. albopictus*	Connecticut and New York, USA	Field	Asian; ECSA-IOL	120 individuals per population	>70% infection beginning at 4 dpi	30–100% after 14 dpi, varying by mosquito population	Up to 27.5%	4 dpi	27:22 °C	RT-qPCR; plaque assay for virus stocks
8	Lutomiah et al. [15]	2021	*Cx. quinquefasciatus*	Mombasa, Kenya	Field	ECSA	Total: 47 individuals; 16 tested at 7 dpi and 14 dpi, 15 at 21 dpi	7 dpi: 25% (4/16); 14 dpi: 31.3% (5/16); 21 dpi: 26.7% (4/15)	7 dpi: 25% (1/4); 14 dpi: 40% (2/5); 21 dpi: 13.3% reported	TE: 7 dpi: 6.3% (1/16); 14 dpi: 12.5% (2/16); 21 dpi: 0%; TR: 100% at 7 and 14 dpi	7 dpi	28 °C	RT-PCR/qPCR and CPE/virus isolation
9	Rodrigues et al. [8]	2021	*Ae. aegypti*	Brazil (Belo Horizonte)	Field	ECSA	600 individuals (40 per each of the 15 experimental groups)	100% for all single and multiple infections	100% for all single and quadruple infections; 90% for CHIKV in CDY triple infection	100% in single and quadruple infections; varied from 40% to 100% in dual and triple infections	14 dpi	28 °C	RT-PCR/qPCR
10	Phumee et al. [16]	2021	*Cx. quinquefasciatus*	Bangkok, Thailand	Both	ECSA-IOL	Total: 43 total (16 males, 27 females)	29.7% (8/27)	NA	NA	NA	28 ± 2 °C	Nested RT-PCR; virus isolation/CPE; immunofluorescence assay
11	Jansen et al. [58]	2021	*Ae. koreicus*	Western Germany	Field	ECSA	24 ± 5 °C: 34; 27 ± 5 °C: 151	24 ± 5 °C: 17.6% (6/34); 27 ± 5 °C: 68.2% (103/151)	NR	24 ± 5 °C: 0% (0/34); 27 ± 5 °C: 4.6% (7/151); TR at 27 ± 5 °C: 6.8% (7/103)	14 dpi	24 ± 5 °C; 27 ± 5 °C	RT-qPCR; cell culture/CPE; plaque assay
12	Calvez et al. [59]	2022	*Ae. aegypti*	Vientiane Capital, Lao PDR	Field	Asian; ECSA-IOL	28–30 females per group/time point	ECSA-IOL: 7% at 3 dpi to 38% at 14 dpi; Asian: 50% at 3 dpi, 33% at 7 dpi, 53% at 14 dpi	ECSA-IOL: 0% at 3 dpi, 83% at 7 dpi; 64% at 14 dpi; Asian: 50% at 3 dpi, 100% at 7 dpi, 75% at 14 dpi	Low TE: positive values from 3% for ECSA-IOL at 7 dpi to 7% for Asian at 14 dpi; TR ranges across 14–20% where saliva-positive	Asian: 3 dpi; ESCA-IOL: 7 dpi	30 ± 2 °C	RT-PCR/qPCR; CPE on Vero E6 cells for infectious saliva particles
13	Terradas et al. [60]	2023	*An. albimanus*	El Salvador	Lab	Asian	Total: 90 30 mosquitoes per time point	33.3% at 7 dpi; 10% at 10 dpi; 10% at 14 dpi	6.7% at 7 dpi; 0% at 10 and 14 dpi	0%	NA	27 ± 1 °C	Focus-forming assay; forced salivation
14	Azman et al. [61]	2024	*Ae. aegypti*	Malaysia	Both	ECSA-IOL	20 mosquitoes per time point/ condition	Midgut infection by virus titration: MC1 0–100%; KL 15–100%; oral infection at 7 dpi by PCR/culture: MC1 15–90%/0–75%; KL 45–100%/15–100%	Heads/thoraxes dissemination by virus titration: MC1 0–35%; KL 0–85%; MC1 pCMV-p2020A 35% vs. pCMV-p2020V 5% at 3 dpi	NR	NR	28 ± 1 °C	Real-time PCR; virus titration/TCID50
15	Bohers et al. [46]	2024	*Ae. albopictus*	Paris, France	Field	ECSA-IOL	Total: 273 individuals exposed across time; 30 tested at 7 dpi for peak values	7 dpi: 63.3% (19/30)	7 dpi: 89.5% (17/19)	TE: 7 dpi: 20.0% (6/30)	7 dpi	28 °C	FFU titration on *Ae. albopictus* C6/36 cells
16	Lühken et al. [9]	2024	*Ae. albopictus*	Germany	Lab	ECSA-IOL	177 individuals	100%	NR	15 °C: 5.56% (1/18); 15 ± 5 °C: 32.5% (13/40); 18 °C: 50.0% (16/32); 18 ± 5 °C: 54.3% (19/35); 21 °C: 39.1% (9/23); 21 ± 5 °C: 58.6% (17/29)	14 dpi	15 °C, 15 ± 5 °C, 18 °C, 18 ± 5 °C, 21 °C, 21 ± 5 °C	RT-qPCR; infectious saliva/cell-culture assay
17	Anyango et al. [62]	2025	*Ae. aegypti*	Kenya (Kisumu and Busia counties)	Field	ECSA	260 individuals	Overall: 56.5% (147/260) Busia: 57.8% (78/135); Kisumu: 55.2% (69/125)	Overall: 78.2% (115/147) Busia: 71.8% (56/78); Kisumu: 85.5% (59/69)	Overall: 26.1% (30/115) Busia: 25.0% (14); Kisumu: 27.1% (16)	5 dpi	28 ± 1 °C	Virus isolation/culture; plaque/focus assay
18	Jansen et al. [63]	2025	*Ae. japonicus*	Germany	Field	ECSA	33 at 21 ± 5 °C; 38 at 24 ± 5 °C; 38 at 27 ± 5 °C	21 ± 5 °C: 81.8% (27/33); 24 ± 5 °C: 81.6% (31/38); 27 ± 5 °C: 89.5% (34/38)	NR	TE: 0% at 21 ± 5 °C & 24 ± 5 °C; 2.9% (1/34) at 27 ± 5 °C	14 dpi	21 ± 5 °C, 24 ± 5 °C, 27 ± 5 °C	RT-qPCR; cell culture/plaque assay for infectious saliva
19	Sanon et al. [64]	2025	*Ae. aegypti*	Ouagadougou, Burkina Faso	Field	West African	NR	Urban: 35%; Peri-urban: 0%	Urban: 85%; Peri-urban: 41–45%	TE: 14–18%	7 dpi	28 ± 1 °C	Plaque assay/TCID50/CPE-based titration
20	Hafsia et al. [65]	2025	*Ae. aegypti*; *Ae. albopictus*	Southwestern Indian Ocean (Seychelles, Comoros, Reunion, Mayotte)	Both	ECSA-IOL	19–48 specimens per mosquito line/time point for saliva collection	*Ae. aegypti*: >90% at 7 and 14 dpe; *Ae. albopictus*: high susceptibility across lines	*Ae. aegypti*: 90.8% *Ae. albopictus*: 72.7% at 7 dpe to 88.8% at 14 dpe; *Ae. Aegypti*: 90.8%	TE: maximum 62.5% *Ae. aegypti*: 90.8%; *Ae. albopictus*: 11.0% at 7 dpe to 23.8% at 14 dpe	7, 14 dpi	28 °C	Plaque assay
21	Visser et al. [47]	2025	*Ae. aegypti*	The Netherlands	Lab	West African	10–40 mosquitoes per treatment/time point	Single infection: 94%; Dual/triple coinfection did not significantly change except lower total load in triple infection	NR	12%	10 dpi	28 °C, 70% RH	RT-qPCR/qPCR; cell culture/CPE for saliva infectivity
22	Gutierrez-Bugallo et al. [66]	2026	*Ae. aegypti*	Havana, Cuba	Both	Not reported	34 mothers exposed, 12 mothers at 2nd gonotrophic cycle, and 72 daughter saliva samples	Vertical transmission study: infected mothers at 2nd gonotrophic cycle: 12/12; Daughters with infected saliva: 11/72	NR	Filial infective rate in saliva (FIR-S): 15% (11/72); vertical transmission rate in saliva (VTR-S): 50% (7/12 families)	0 dpi/immediate upon emergence	28 °C	RT-qPCR; plaque assay; NGS/sequencing

NR = not reported; NA = not applicable. IR = infection rate; DR = dissemination rate; TE = transmission efficiency; TR = transmission rate; MIR = minimum infection rate; EIP = extrinsic incubation period; dpi = days post-infection; dpe = days post-exposure. MC1 = *Aedes aegypti* field strain from Petaling Jaya, Selangor; KL = *Aedes aegypti* laboratory colony from Kuala Lumpur; pCMV-p2020A and pCMV-p2020V = infectious clones of Malaysian ECSA-IOL CHIKV isolate MY/2020/3092435; CDY = CHIKV/DENV/YFV triple-coinfection group.

The experimental conditions under which these laboratory studies were conducted are summarised in Table 3. These parameters varied substantially across studies: infectious blood-meal titres ranged from approximately 10^5 to 10^9 infectious units per millilitre; mosquitoes were exposed most often through artificial membrane feeders (for example, the Hemotek system) but also by droplet feeding or, in one study, by intrathoracic inoculation; and transmission was assessed predominantly by forced salivation combined with plaque, focus-forming, or cytopathic-effect assays, with one study additionally employing an in vivo mouse-transmission model. This methodological heterogeneity in infectious dose, feeding route, incubation temperature, and saliva assay directly affects the measured infection, dissemination, and transmission outcomes, underscoring the descriptive rather than pooled treatment of the vector competence values reported in Section 4.

## 4. Species-Level Vector Evidence

Mosquito species were interpreted using two complementary evidence types: field detection and laboratory vector competence. Field detection indicates that either CHIKV RNA or the virus was detected in naturally collected mosquitoes, whereas laboratory vector competence studies assess whether experimentally exposed mosquitoes can support infection, dissemination, and transmission. This distinction is important, because PCR positivity alone does not confirm vector status. Throughout this review, we therefore distinguish three levels of evidence that are often conflated: the detection of CHIKV RNA, which signals exposure only; isolation of the infectious virus, which confirms viability of the virus; and demonstrated transmission under experimental or in vivo conditions, which is required to establish vector competence.

Based on the reviewed studies, mosquito species were grouped into principal vectors, potential regional or emerging vectors, field-positive but unconfirmed vectors, and species with weak or inconsistent evidence of vector competence (Table 4). This classification avoids overinterpretation of RNA-only detection while still identifying mosquito taxa that may warrant further study. This classification reflects the 2020–2026 evidence base and is not intended as an exhaustive catalogue of all historical vector records; earlier experimental findings, notably the adaptation of CHIKV to *Ae. albopictus* through the E1-A226V substitution [10] and the demonstrated competence of Kenyan *Ae. vittatus* populations [67], are considered where they inform interpretation of these groupings.

### 4.1. Main CHIKV Vectors

*Aedes aegypti* was the most frequently detected species in the field surveillance and remains the predominant urban CHIKV vector. Its role is linked to its close association with humans, artificial-container breeding, domestic resting behaviour, and anthropophilic feeding [68]. Laboratory studies also strongly support its role, showing that *Ae. aegypti* can support the infection, dissemination, and transmission of Asian, ECSA, ECSA-IOL, and West African genotype groups (Table 2).

On the other hand, *Ae. albopictus* is the second major vector identified in this review and is documented in both field surveillance and laboratory datasets, where it was reported across diverse settings, including Brazil, Central Africa, Ecuador, Colombia, Ghana, and China (Table 1 and Table 2). Its ecological flexibility, ability to occupy peri-urban and vegetated environments, adaptation to cooler climates [69], and competence for ECSA and ECSA-IOL strains (Table 1 and Table 2) underscore its role as a vector where this species is either established or expanding. Within Europe and the Mediterranean, this competence is well-documented: *Ae. albopictus* populations from Germany and Paris transmitted ECSA-IOL CHIKV, even at the lower temperatures characteristic of temperate summers [9,46]; a study from the Netherlands confirmed *Ae. aegypti* competence for a West African strain [47]; and earlier experimental work showed that Lebanese *Ae. albopictus* transmitted CHIKV in saliva in approximately 30% of mosquitoes at ten days post-infection [45], with the last predating the review window and therefore outside the tabulated dataset.

Vector competence in both principal vectors varied across mosquito population, virus genotype, infectious dose, incubation temperature, extrinsic incubation period, and assay method. Therefore, local *Ae. aegypti* and *Ae. albopictus* populations should ideally be evaluated using locally circulating CHIKV strains under ecologically relevant temperature conditions.

### 4.2. Potential Regional and Emerging Vectors

Several other *Aedes* species have shown possible regional relevance (Table 4). *Ae. vittatus* yielded confirmed CHIKV detections in only one field study in Kenya [28] (Table 1); the other surveillance studies that screened it detected no CHIKV. Although no 2020–2026 laboratory records were included in the dataset, earlier work has shown the competence of Kenyan *Ae. vittatus* populations for ECSA-lineage CHIKV [67], suggesting possible regional importance that requires an updated evaluation using contemporary strains and local populations.

Moreover, *Aedes polynesiensis* (Marks, 1951) can become infected with CHIKV under experimental conditions [53], which may be relevant in Pacific Island settings, where local mosquito communities and human–vector contact patterns differ from continental settings. In addition, laboratory studies have shown that *Aedes koreicus* (Edwards, 1917) and *Aedes japonicus* (Theobald, 1901) can become infected with CHIKV under experimental conditions, although transmission outcomes were low or inconsistent (Table 2). This limited competence evidence is relevant, as both species are invasive *Aedes* mosquitoes with expanding distributions in temperate regions. Therefore, these species should be considered emerging or uncertain vectors.

Other field-detected *Aedes* species with confirmed CHIKV positivity included *Ae. fluviatilis* [21] and *Ae. luteocephalus* [38]. These detections were suggestive of possible exposure during active transmission, but neither has been sufficiently evaluated through laboratory vector competence assays (Table 1).

The epidemiological weight of field detection also depends on the feeding ecology of the species involved, as not all culicids implicated above are strictly anthropophilic. *Ae. aegypti* is highly anthropophilic and endophilic, feeding preferentially and repeatedly on humans in and around dwellings, which maximises its capacity to acquire and transmit CHIKV during urban outbreaks [70]. *Ae. albopictus* is a more opportunistic, catholic feeder with pronounced zoophilic tendencies, biting humans as well as other mammals and birds; this mixed host use lowers its per-bite human transmission efficiency but positions it as a bridge between peri-domestic and sylvatic cycles [71]. Several other field-detected species, including *Ae. vittatus* and forest-associated *Aedes*, together with the largely ornithophilic or mammalophilic *Culex* and *Anopheles* species, feed only opportunistically on humans, so their detection with CHIKV is more plausibly a marker of ecological exposure than of a primary role in human transmission [72]. Feeding preference should therefore be weighed alongside laboratory competence when interpreting the significance of any field-positive species.

Beyond intrinsic competence, the epidemiological plausibility of a species acting as a local vector is strengthened when it is both abundant and CHIKV-positive while in the same place and time as human cases. Several of the field records reviewed here (for example, the outbreak-associated detections in Brazil, Kenya, and Burkina Faso) derive their weight from this exact spatial and temporal coincidence between vector presence and active transmission [72]. Such epidemiological association does not replace experimental confirmation, but it provides an important complementary line of evidence for prioritising species and settings for vector competence testing.

### 4.3. Field-Positive but Unconfirmed Non-Aedes Mosquitoes

CHIKV RNA was detected in several non-*Aedes* species, especially *Cx. quinquefasciatus* and other *Culex* species (Table 1). These detections may reflect recent blood feeding on viraemic hosts, persistence of non-replicating viral RNA, or infections that fail to disseminate beyond the midgut. The current evidence is insufficient to classify *Culex* mosquitoes as principal CHIKV vectors.

In addition, *Anopheles* mosquitoes, including *An. maculipennis* s.l. in field surveillance and *Anopheles albimanus* (Wiedemann, 1820) in laboratory studies, showed limited or inconsistent evidence of vector competence, with occasional experimental infection but no consistent demonstration of transmission (Table 2). Laboratory findings indicate that while some *Anopheles* mosquitoes may become infected, transmission has not yet been consistently demonstrated. Interestingly, *Psorophora*, *Wyeomyia*, and other culicids were mainly reported from broader Brazil field surveillance studies and are best interpreted as evidence of ecological exposure rather than confirmed transmission roles (Table 1).

### 4.4. Descriptive Comparison of Infection, Dissemination, and Transmission Rates

As the laboratory vector competence studies differed in mosquito population origin, CHIKV strain, infectious dose, incubation temperature, extrinsic incubation period, sample size, detection method, infection rate, dissemination rate, and transmission efficiency were compared descriptively rather than pooled statistically. This approach allowed for broad comparison of vector competence patterns across mosquito species and CHIKV genotype groups while avoiding overinterpretation of heterogeneous experimental designs and known sources of mosquito infection rate errors [7,13,14,52,60].

**Figure 3:**
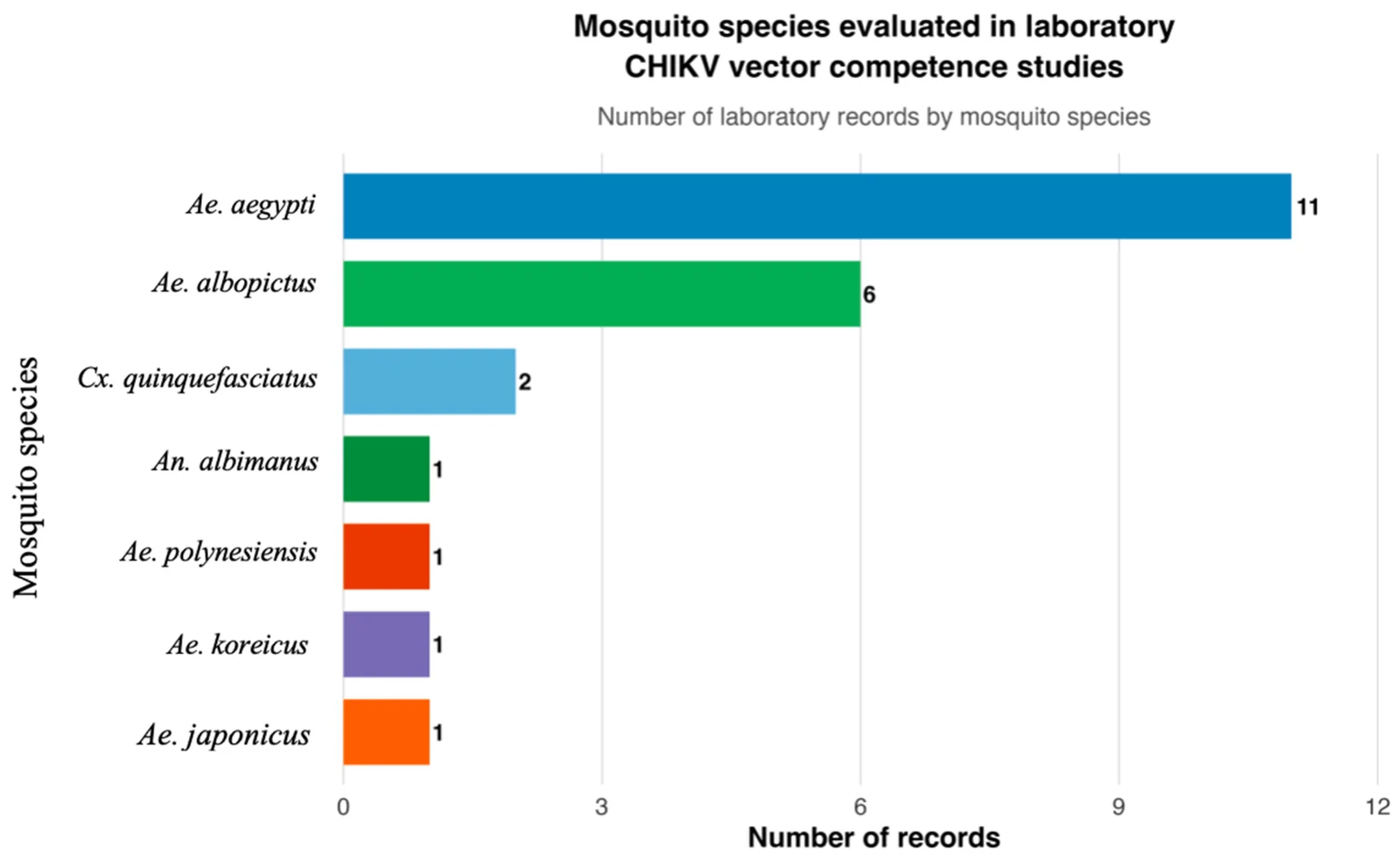
Mosquito species evaluated in laboratory CHIKV vector competence studies (*n* = 22).

**Figure 4:**
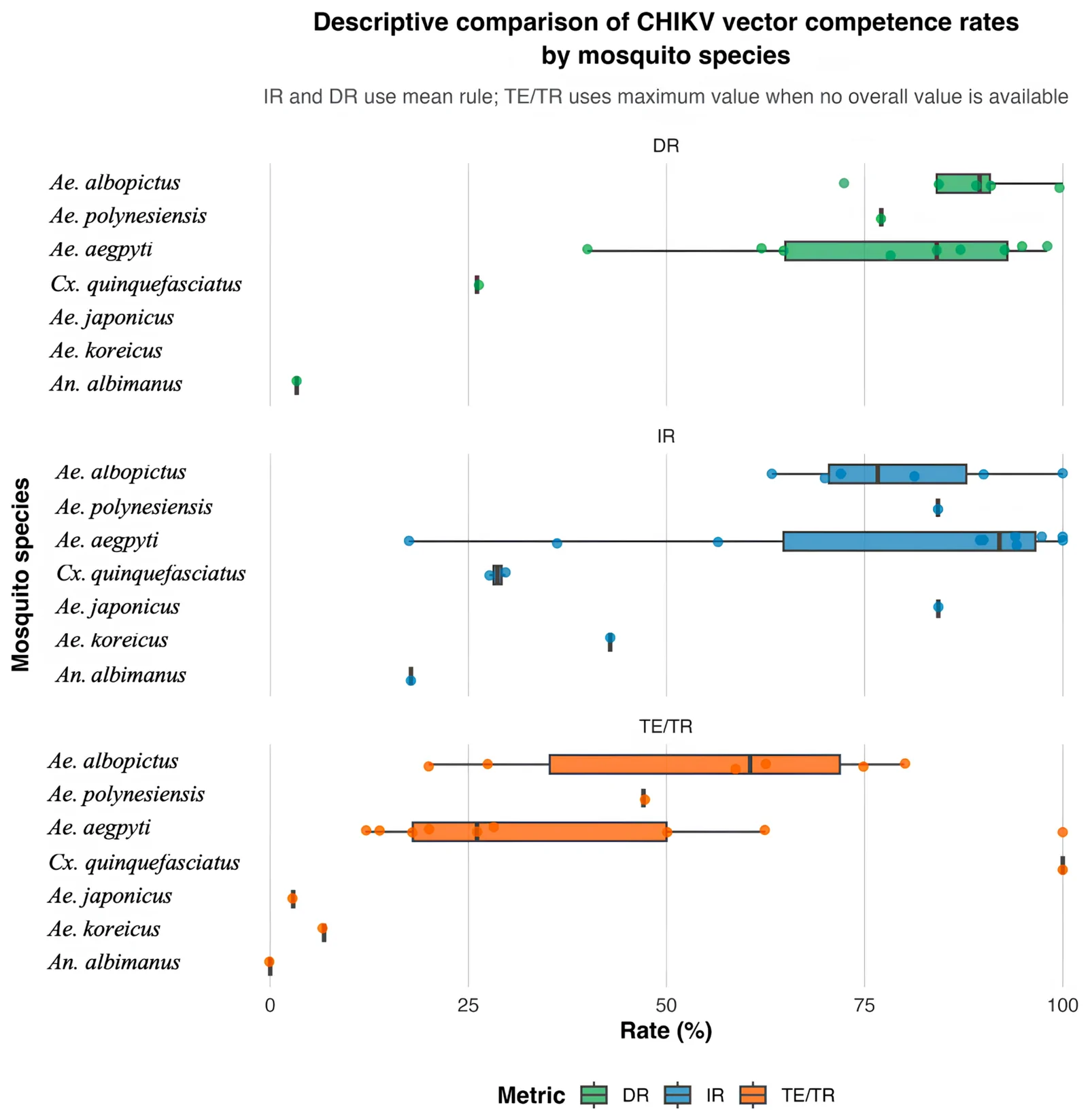
Descriptive comparison of infection rates (IRs), dissemination rates (DRs), and transmission efficiency/rates (TE/TRs) by mosquito species in laboratory vector competence studies. Values are descriptive summaries rather than pooled meta-analytic estimates.

Across the laboratory studies, *Ae. aegypti* and *Ae. albopictus* showed the most consistent evidence of CHIKV susceptibility and transmission potential (Table 5). The range of mosquito species evaluated in laboratory vector competence studies is shown in Figure 3, which highlights the predominance of the two principal *Aedes* vectors among the experimentally tested species. *Ae. aegypti* was represented in 11 laboratory studies. The calculated mean infection rate was approximately 76%, ranging from 17.5% to 100%. Dissemination data were available in nine studies, with a mean DR of approximately 77% and a range of 25% to 98%. Transmission efficiency (TE), or transmission rate (TR), was reported in nine studies, with a mean of approximately 37%, ranging from 12% to 100%. These findings indicate that *Ae. aegypti* is generally highly susceptible to CHIKV infection and is frequently capable of supporting viral dissemination under experimental conditions (Table 2 and Figure 4). 

*Aedes albopictus* showed similarly strong vector competence patterns. Among six laboratory studies, all six provided IR data, with a mean infection rate of approximately 79% and a range of 63.3% to 100%. Furthermore, DR was available in five studies, with a mean of approximately 80% and a range of 65% to 90.8%. On the other hand, TE/TR was reported in six studies, with a mean of approximately 47% and a range of 20% to 80%. Compared with *Ae. aegypti*, *Ae. albopictus* showed slightly higher mean infection and transmission values in the dataset, whereas the dissemination values were similar. However, this comparison should be interpreted with caution, as the studies differed in terms of virus strain, mosquito origin, incubation period, and experimental design. Nevertheless, the data support *Ae. albopictus* as a highly competent CHIKV vector, particularly in settings where ECSA or ECSA–Indian Ocean lineage strains are circulating (Table 2, Figure 4).

When the same descriptive values are considered by virus lineage rather than by species alone, a consistent pattern emerges that reflects genotype-by-vector compatibility. ECSA and ECSA-IOL strains, several of which carry the E1-A226V substitution, were associated with the highest transmission efficiencies recorded in the dataset, particularly in *Ae. albopictus* (for example, ECSA-IOL transmission efficiency peaked around 55% in Tunisian *Ae. albopictus* with elevated dissemination in European populations), whereas Asian-genotype infections of *Ae. aegypti* more often produced high infection and dissemination rates but comparatively low infectious saliva transmission (Table 2). West African strains were represented by only a few *Ae. aegypti* records with modest transmission. A fully quantitative genotype-stratified analysis would be constrained by incomplete genotype reporting and small per-genotype sample sizes (a limitation noted in Section 6); therefore, the values in Table 5 are presented by species, with the genotype indicated per study in Table 2, while the genotype contrasts mentioned above should be read as descriptive rather than statistical. Even so, this pattern reinforces that the compatibility between circulating lineage and locally abundant vectors, rather than by either factor alone, governs outbreak potential in a given region.

Other mosquito species showed more variable or limited evidence. *Culex quinquefasciatus* was represented in two laboratory studies, with IR values averaging approximately 29%. Dissemination data were limited, with only one study reporting a dissemination rate of 26.1%. Transmission efficiency or transmission rate was extractable from only one study, with maximum values reaching 100%. Although these findings indicate that CHIKV detection or limited transmission-related outcomes may occur under certain experimental conditions, the limited number of studies and inconsistent evidence currently provide insufficient support to establish *Cx. quinquefasciatus* as an epidemiologically important CHIKV vector.

Among other experimentally evaluated *Aedes* species, *Ae. polynesiensis* showed relatively high IR of 84.25%, DR of 77.1%, and TE/TR of 40.7% in the available laboratory records. This suggests that *Ae. polynesiensis* may be competent for CHIKV under experimental conditions and may have regional importance, particularly in Pacific Island settings. *Aedes japonicus* also showed a high IR of 84.3%, but the reported TE/TR was very low at 2.9%, indicating that infection alone did not translate into efficient transmission. *Aedes koreicus* showed a moderate infection rate of 42.9% and low maximum TE/TR of 6.8%, suggesting limited transmission potential based on the available data. *Anopheles albimanus* showed low IR and DR, with rates of 17.77% and 3.35%, respectively. In addition, there was no transmission detected. These findings support the interpretation that not all mosquito species with detectable infection are epidemiologically important vectors.

## 5. Detection and Diagnostic Methodologies Used in CHIKV Mosquito Studies

### 5.1. Molecular Detection

RT-PCR and qPCR were the dominant methods used in field surveillance studies. These methods are suitable for screening mosquito pools and are considered a gold standard for detecting the RNA of arboviruses, showing high efficiency due to its sensitivity and specificity [30,73]. These are especially useful during outbreaks because they can detect CHIKV RNA even when viral loads are low.

However, molecular detection does not confirm virus infection or vector competence. A mosquito may test positive because of a recent blood meal, residual viral RNA, a non-infectious virus, or an abortive infection [74]. Therefore, PCR-positive field results should be interpreted as confirmation of CHIKV detection rather than as direct evidence of transmission.

### 5.2. Virus Isolation and Infectivity Assays

Virus isolation, plaque assay, focus-forming assay, and cell culture-based methods provide stronger evidence because they indicate viable or infectious viruses. These methods were less common in field surveillance studies but were more frequently used in laboratory vector competence studies [75].

In laboratory studies, infectious virus detection in saliva is particularly important because it directly supports transmission potential. Infection rates and dissemination rates are useful but are less definitive than transmission efficiency [65]. Therefore, laboratory studies that include saliva testing provide stronger evidence for vector competence than studies that assess only body infections (Table 2). Beyond saliva assays, in vivo transmission models offer the most direct confirmation of competence, as experimentally, CHIKV-infected *Ae. albopictus* has transmitted the virus to naive mice, who subsequently developed viraemia and arthritis [76], while within the present dataset, another study similarly confirmed transmission using an in vivo mouse model [56]. Such animal-based transmission assays remain uncommon but can provide valuable corroboration of saliva-based vector competence metrics.

### 5.3. Sequencing and Genomic Approaches

Sequencing and next-generation sequencing were used in a smaller number of studies. These approaches are important because they allow for genotype confirmation, outbreak lineage identification, and phylogenetic analysis [77]. The limited use of sequencing contributed to incomplete genotype reporting in field studies (Table 1).

Future field surveillance should incorporate sequencing of CHIKV-positive mosquito pools whenever possible, especially during outbreaks. This would allow for stronger linkage between field detection, virus lineage, and vector species.

## 6. Limitations

This review has several limitations. Firstly, the literature search was conducted using PubMed and Google Scholar; therefore, some of the regional reports, grey literature, and non-indexed studies may have been missed. In addition, the included studies were heterogeneous in terms of design, mosquito species, sample size, virus strain, diagnostic method, and reporting format.

Many field studies did not report the CHIKV genotype. This limits the analysis of lineage-specific vector associations and regional viral movements. Other than that, most field studies relied on molecular detection without virus isolation. Furthermore, the infection metrics were inconsistently reported across studies. Finally, several non-*Aedes* and other secondary mosquito species were reported as CHIKV-positive in the field but lacked laboratory confirmation.

These limitations precluded meta-analysis and required a descriptive synthesis. This is important, as mosquito infection estimates can be biassed when pool sizes, sampling intensity, collection timing, and diagnostic sensitivity differ across studies. Even so, the combined evidence provides a useful overview of current CHIKV vector knowledge and identifies priorities for future surveillance and experimental work.

## 7. Integrated Surveillance and Control of CHIKV Vectors

**Figure 5:**
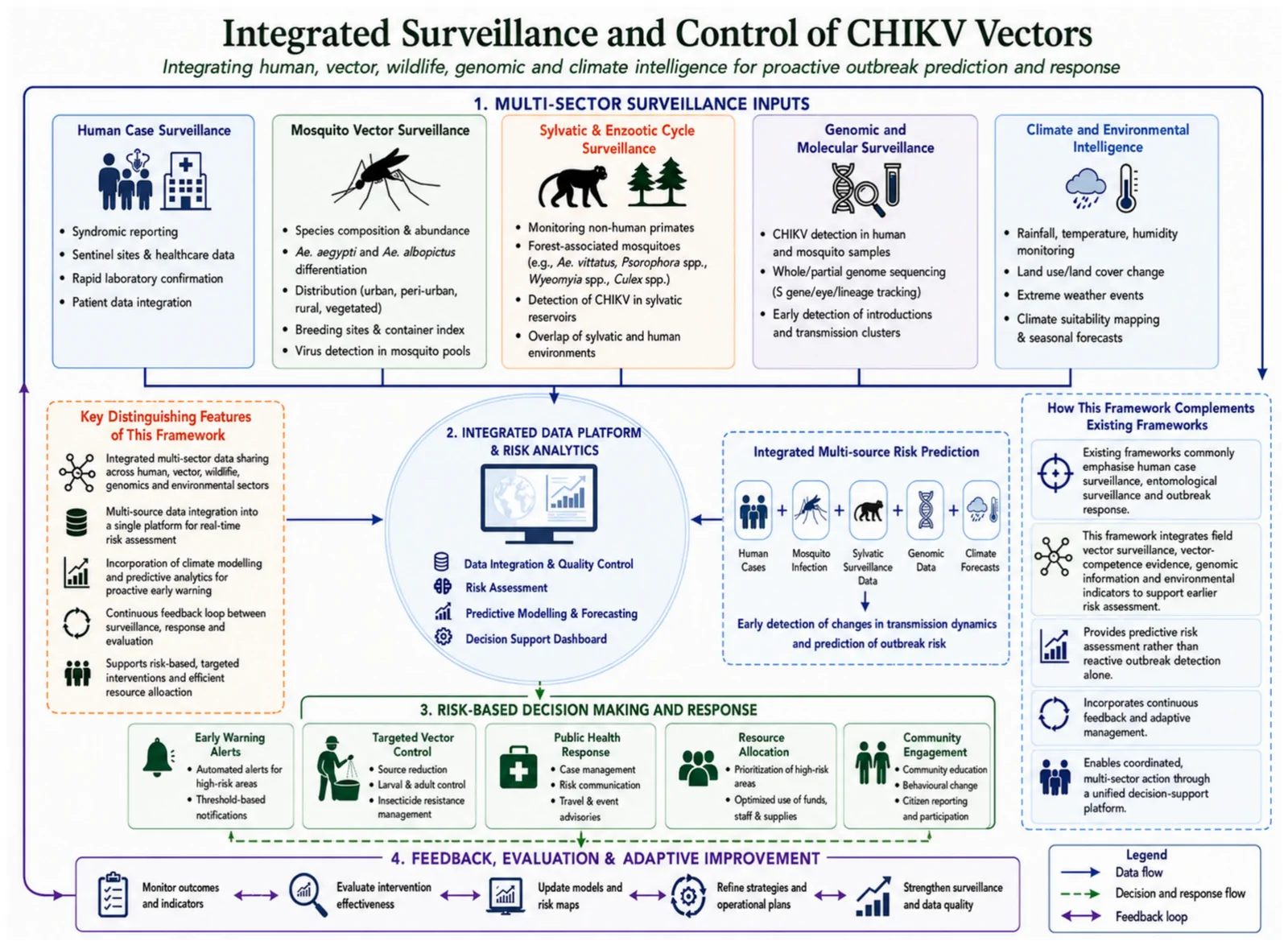
Proposed framework for integrated surveillance and control of CHIKV vectors.

Although CHIKV is primarily recognised as a human arboviral disease, its transmission dynamics are shaped by interactions among humans, mosquitoes, animals, and the environment. Effective preparedness therefore depends on the integration of surveillance and controls to link human-health surveillance, vector surveillance, environmental management, climate monitoring, urban planning, and community-level prevention. A comprehensive integrated framework for CHIKV surveillance and controls is proposed (Figure 5), linking mosquito biology, human infections, environmental conditions, and multi-sectoral activities. The individual components of Figure 5 are not intended to constitute an entirely new surveillance system. Many are already included, to varying degrees, in established arboviral surveillance, integrated vector management, and One Health strategies. For example, existing frameworks commonly emphasise human case detection, laboratory confirmation, entomological surveillance, source reduction, and insecticide-based vector control. The framework proposed here complements these approaches by bringing together the specific evidence synthesised in this review within a single CHIKV-focused structure. In particular, it links the field detection of CHIKV in mosquito populations with the laboratory evidence of vector competence, viral genotype information, environmental and climate indicators, urban infrastructure, and community-level prevention. It also highlights the need for information to move continuously from surveillance to risk assessment, targeted intervention, and subsequent evaluation. This integrated evidence-to-action pathway represents the main distinguishing feature of Figure 5, instead of considering any single surveillance component in isolation. The proposed framework is therefore intended to complement, rather than replace, existing integrated approaches, including the One Health control strategy proposed by Fang et al. [78].

The reviewed evidence highlights the role of mosquito vectors as the central link between environmental conditions and human infection. *Aedes aegypti* is closely associated with human dwellings, artificial containers, poor water storage practices, and dense urban settlements [68]. These characteristics make CHIKV transmission strongly dependent on human behaviour and the built environment. In contrast, *Ae. albopictus* can occupy peri-urban, rural, vegetated, and temperate habitats, linking CHIKV risk to broader environmental and ecological changes [68,69]. Therefore, vector control cannot rely solely on insecticide application but must also include environmental sanitation, removal of breeding containers, water management, community engagement, and urban planning to reduce mosquito breeding opportunities.

Climate and environmental changes are also important drivers of CHIKV risk. Temperature, rainfall, humidity, and land-use changes influence mosquito abundance, survival, biting activity, and viral replication [78]. Laboratory vector competence studies show that temperature and extrinsic incubation period can affect infection, dissemination, and transmission outcomes. Warmer conditions may shorten the extrinsic incubation period and increase the chance that mosquitoes become infectious within their lifespan, while changes in rainfall may create more larval habitats [79]. These factors can expand the geographical range and seasonal window of CHIKV transmission, particularly in areas where *Ae. albopictus* and other invasive mosquitoes are established. Modelling under climate change scenarios projects poleward shifts in *Aedes*-borne transmission risks, with optimal temperature ranges of 21.3–34.0 °C for *Ae. aegypti* and 19.9–29.4 °C for *Ae. albopictus*, as well as the potential exposure of nearly a billion additional people by 2080 (Table 2).

Urban transmission is mainly driven by human–*Aedes*–human cycles involving *Ae. aegypti* and *Ae. albopictus*. However, in some regions, especially in Africa, CHIKV may also be maintained in sylvatic or enzootic cycles involving non-human primates and forest-associated mosquitoes [78,80]. The detection of CHIKV RNA in non-*Aedes* and other secondary mosquito species, such as *Ae. vittatus*, *Psorophora* spp., *Wyeomyia* spp., *Culex* spp., and other culicids, suggests that mosquito exposure may occur beyond strictly urban vectors. Although these detections do not confirm vector competence, they indicate that broader ecological surveillance may be necessary in areas where human settlements overlap with forest-associated, peri-domestic, or wildlife-associated habitats. In addition, a full treatment of enzootic maintenance, non-human primate reservoirs, and the vector ecology of forest-associated *Aedes* (for example, *Ae. furcifer* and *Ae. luteocephalus*) would require dedicated sylvatic surveillance, and remains a priority for future work.

Therefore, integrated surveillance is essential to monitor the potential outbreaks of CHIKV. Human case surveillance alone may detect outbreaks only after transmission is already established [81]. Mosquito surveillance can provide earlier warning of viral circulation, particularly when combined with molecular detection, virus isolation, sequencing, and vector abundance monitoring. Environmental surveillance, such as monitoring rainfall patterns, temperature anomalies, land-use changes, and mosquito breeding habitats, can help identify areas at increased risk. Together, these data streams can support more timely and targeted interventions.

Integrated CHIKV control also depends on collaboration across sectors. Public health authorities, entomologists, veterinarians, environmental scientists, urban planners, local governments, and communities all play important roles in CHIKV prevention. Public health teams are needed to detect and report human cases. Entomologists are required to identify vector species, assess vector abundance, and evaluate insecticide resistance. Beyond taxonomic identification, entomologists are also responsible for determining vector hotspots, actively collecting vectors in the field, and instructing and supervising mosquito-control operators in the treatment of breeding sites and resting mosquitoes. Environmental and municipal sectors are needed to manage waste, water storage, drainage, and urban breeding sites. Community participation is also essential, because many *Aedes* breeding habitats occur in household or peri-household environments.

The findings of this review support the need for integrated CHIKV preparedness. Surveillance should prioritise confirmed vectors, such as *Ae. aegypti* and *Ae. albopictus*, but broader mosquito sampling may be useful during outbreaks or in ecologically complex settings. Positive mosquito pools should be subjected to virus isolation and sequencing whenever possible to determine whether infectious viruses are present and which genotype is circulating. Climate and environmental data should be integrated with entomological and epidemiological data to better predict outbreak risk.

Overall, the proposed framework differs from surveillance approaches that treat clinical, entomological, environmental and control activities as largely separate operational streams by explicitly connecting them within a continuous cycle of data integration, risk assessment, response, and evaluation. Its contribution lies in synthesising the evidence reviewed here into a CHIKV-specific, multi-sector structure that can support both routine preparedness and outbreak response. Such coordination is increasingly important as urbanisation, climate change, global travel, and vector expansion alter the geographical landscape of CHIKV transmission. The movement of viraemic travellers, the international trade in used tyres associated with the dispersal of *Ae. albopictus*, and the continuing establishment of this vector in temperate regions create pathways for cross-border transmission. The introduction of compatible CHIKV variants into areas with established and competent *Ae. albopictus* populations therefore represents an important risk that internationally coordinated surveillance should address. Recent autochthonous transmission in European settings further demonstrates that surveillance should integrate imported human cases, local vector distributions, viral genotype information, and environmental suitability instead of considering these indicators independently.

## 8. Conclusions and Future Directions

This review identifies *Ae. aegypti* and *Ae. albopictus* as the principal CHIKV vectors, as supported by repeated field detections and laboratory vector competence evidence. *Aedes aegypti* remains central to urban transmission, whereas *Ae. albopictus* is important due to its ecological flexibility and continuing expansion into peri-urban and temperate environments.

Other *Aedes* species, including *Ae. vittatus*, *Ae. polynesiensis*, *Ae. koreicus*, and *Ae. japonicus*, may have regional or emerging relevance but require further validation. Although *Ae. vittatus* has demonstrated laboratory competence in East African populations [67], its role in West Africa and other regions remains insufficiently defined. CHIKV RNA detection in *Culex*, *Anopheles*, *Psorophora*, *Wyeomyia*, and other culicids indicates broader mosquito exposure, but it does not confirm vector competence [42,60,82].

Future CHIKV vector studies should integrate field surveillance with laboratory vector competence assays. Field-positive mosquito pools should be prioritised for virus isolation and sequencing to confirm viral viability, genotypes, and epidemiological relevance. Laboratory studies should use local mosquito populations and locally circulating CHIKV strains whenever possible, and should assess infection, dissemination, and infectious saliva production rather than relying only on infection positivity.

Standardised reporting is also essential. Future studies should clearly report the mosquito species, collection sites, sample sizes, pool sizes, infection rates, minimum infection rates, virus genotypes, detection methods, extrinsic incubation periods, incubation temperatures, and transmission efficiency. Minimum data standards for vector competence experiments, such as those proposed by Wu et al. [83], would improve comparability across CHIKV mosquito studies.

Overall, future CHIKV preparedness requires an integrated framework that brings together human case surveillance, mosquito monitoring, environmental risk assessment, genomic sequencing, community participation, and standardised vector competence assays. This integrated approach would clarify mosquito vector roles, strengthen early-warning systems, and improve targeted prevention of future CHIKV outbreaks.

## Figures and Tables

**Table 3:** Experimental exposure and saliva assay parameters for the laboratory vector competence studies listed in Table 2.

No.	Study	Mosquito Species	Infectious Blood-Meal Titre	Feeding/Exposure Method	Saliva Collection/Transmission Assay
1	Robison et al. 2020 [52]	*Ae. aegypti*	9 × 10^6 PFU/mL	Water-jacketed glass feeder; hog-gut membrane; defibrinated calf blood	Forced salivation; plaque assay
2	Bohers et al. 2020 [7]	*Ae. albopictus*	10^7 FFU/mL	Hemotek feeder	Forced salivation; focus-forming assay
3	Calvez et al. 2020 [53]	*Ae. polynesiensis*	10^7 PFU/mL	Hemotek feeder	Forced salivation; plaque assay
4	Shin et al. 2020 [54]	*Ae. aegypti*	6.82 log10 PFU/mL	Hemotek feeder	Not assessed
5	Gutiérrez-Bugallo et al. 2020 [55]	*Ae. aegypti*	10^7 TCID50/mL	Hemotek feeder	Forced salivation; FFU/plaque assay
6	Kaczmarek et al. 2020 [56]	*Ae. albopictus*	1–4 × 10^6 (high)/2–7.5 × 10^5 (low) IVP/mL	Hemotek feeder	Forced salivation; in vivo mouse transmission
7	Gloria-Soria et al. 2020 [57]	*Ae. albopictus*	7.4 log10 PFU/mL	Hemotek feeder	Forced salivation; plaque assay
8	Lutomiah et al. 2021 [15]	*Cx. quinquefasciatus*	10^8.6 PFU/mL	Hemotek feeder	Forced salivation; virus isolation/plaque
9	Rodrigues et al. 2021 [8]	*Ae. aegypti*	1 × 10^5 PFU	Intrathoracic inoculation; blood-fed on naive mice post-infection	Not assessed
10	Phumee et al. 2021 [16]	*Cx. quinquefasciatus*	9.2 × 10^6 PFU/mL	Artificial feeding apparatus	Not assessed
11	Jansen et al. 2021 [58]	*Ae. koreicus*	10^6 PFU/mL	Two 50 µL blood droplets in vial	Salivation assay; cell culture
12	Calvez et al. 2022 [59]	*Ae. aegypti*	10^6 TCID50/mL	Hemotek feeder	Forced salivation; CPE assay
13	Terradas et al. 2023 [60]	*An. albimanus*	1 × 10^7 FFU/mL	Glass feeder; synthetic membrane; 37 °C	Forced salivation; focus-forming assay
14	Azman et al. 2024 [61]	*Ae. aegypti*	2–6 log10 TCID50/mL	Hemotek feeder	Not assessed
15	Bohers et al. 2024 [46]	*Ae. albopictus*	10^7 FFU/mL	Membrane feeder	Forced salivation; focus-forming assay
16	Lühken et al. 2024 [9]	*Ae. albopictus*	10^6 PFU/mL	Two 50 µL blood droplets in vial	Forced salivation; cell-culture assay
17	Anyango et al. 2025 [62]	*Ae. aegypti*	10^6 PFU/mL	Hemotek feeder	Forced salivation; plaque/focus assay
18	Jansen et al. 2025 [63]	*Ae. japonicus*	10^7 PFU/mL	Two 50 µL blood droplets in vial	Salivation assay; cell culture
19	Sanon et al. 2025 [64]	*Ae. aegypti*	1 × 10^7/1 × 10^8/1.4 × 10^9 TCID50/mL	Oral infection via artificial blood meal	Forced salivation; TCID50/CPE
20	Hafsia et al. 2025 [65]	*Ae. aegypti*; *Ae. albopictus*	10^8 PFU/mL	Hemotek feeder	Forced salivation; plaque assay
21	Visser et al. 2025 [47]	*Ae. aegypti*	2 × 10^7 TCID50/mL target (mean 4.3 × 10^6)	Hemotek feeder	Forced salivation; cell-culture assay
22	Gutiérrez-Bugallo et al. 2026 [66]	*Ae. aegypti*	10^7 TCID50/mL	Hemotek feeder	Forced salivation; plaque assay

**Table 4:** Classification of mosquito species according to the strength of evidence for CHIKV transmission (field detection and laboratory vector competence). Field and laboratory record counts, as well as the studies listed in the authors/reference column, include only the records in which the species was confirmed CHIKV-positive by field detection or was experimentally competent in the laboratory; studies that screened a species without detecting CHIKV are not counted here. The full set of screened studies, including negative results, is compiled in Table 1 and Table 2.

Evidence Category	Mosquito Species/Group	Field Records	Lab Records	Interpretation	Authors/Reference
Principal vectors	*Ae. aegypti*	22	11	Strong field and laboratory evidence; dominant urban vector.	Rodrigues et al. [8]; Lutomiah et al. [15]; Cruz et al. [17]; Teixeira et al. [18]; Almeida-Souza et al. [19]; Banho et al. [20]; de Melo Ximenes et al. [21]; Chand et al. [39]; Munivenkatappa et al. [40]; Vikram et al. [41]; Heath et al. [29]; Laouali et al. [31]; Toé et al. [32]; Abbasi [43]; Carrasquilla et al. [24]; Mantilla-Granados J.S. et al. [25]; Kirstein et al. [26]; Hernández-Acosta et al. [27]; Nwangwu et al. [38]; Da Silva-Neves et al. [48]; Leandro et al. [49]; Silva et al. [50]; Banho et al. [51]; Robison et al. [52]; Shin et al. [54]; Gutiérrez-Bugallo et al. [55]; Calvez et al. [59]; Azman et al. [61]; Anyango et al. [62]; Sanon et al. [64]; Hafsia et al. [65]; Visser et al. [47]; Gutierrez-Bugallo et al. [66]
Principal vectors	*Ae. albopictus*	9	6	Strong vector competence; important peri-urban/rural/temperate vector.	Bohers et al. [7]; Lühken et al. [9]; Banho et al. [20]; de Melo Ximenes et al. [21]; Vairo et al. [33]; Weggheleire et al. [34]; Abbasi [43]; Mantilla-Granados J.S. et al. [25]; Zhou et al. [44]; Da Silva-Neves et al. [48]; Banho et al. [51]; Kaczmarek et al. [56]; Gloria-Soria et al. [57]; Bohers et al. [46]; Hafsia et al. [65]
Potential regional vectors	*Ae. vittatus*	1	0	CHIKV-positive in Kenyan field surveillance [28] and laboratory competent in an earlier Kenyan study [67]; continued field validation and regional population studies are warranted.	Musili et al. [28]
Emerging/ uncertain vectors	*Ae. polynesiensis*	0	1	Laboratory competence; likely regional island relevance.	Calvez et al. [53]
Emerging/ uncertain vectors	*Ae. koreicus*; *Ae. japonicus*	0	2	Experimental susceptibility demonstrated but transmission low or not consistently detected in the laboratory, and no confirmed field vector role: *Ae. koreicus* [58]; *Ae. japonicus* [63].	Jansen et al. [58]; Jansen et al. [63]
Field-positive but unconfirmed	*Culex* spp.; *Anopheles* spp.; *Psorophora* spp.; *Wyeomyia* spp.	*Culex*: 8; *Anopheles*: 1; *Psorophora*: 1; *Wyeomyia*: 1	*Cx. quinquefasciatus*: 2; *An. albimanus*: 1; *Psorophora*/*Wyeomyia*: 0	Field RNA detection indicates ecological exposure; laboratory competence not confirmed (*Cx. quinquefasciatus* [15,16]; *An. albimanus* [60]) and *Psorophora* and *Wyeomyia* were not tested experimentally.	Lutomiah et al. [15]; Phumee et al. [16]; Cruz et al. [17]; Almeida-Souza et al. [19]; Banho et al. [20]; de Melo Ximenes et al. [21]; Bakhshi et al. [42]; Da Silva-Neves et al. [48]; Banho et al. [51]; Terradas et al. [60]

**Table 5:** Descriptive summary of infection rates (IRs), dissemination rates (DRs) and transmission efficiency/rates (TE/TRs) for mosquito species in laboratory CHIKV vector competence studies, 2020–2026. Values are descriptive summaries drawn from the studies in Table 2 (means with observed ranges).

Mosquito Species	IR % Mean (Range)	DR % Mean (Range)	TE/TR % Mean (Range)	No. of Lab Studies
*Ae. aegypti*	~76 (17.5–100)	~77 (25–98)	~37 (12–100)	11
*Ae. albopictus*	~79 (63.3–100)	~80 (65–90.8)	~47 (20–80)	6
*Cx. quinquefasciatus*	~29 (25–31.3)	26.1 (1 study)	TE 6–12.5; TR up to 100	2
*Ae. polynesiensis*	84.3	77.1	40.7	1
*Ae. japonicus*	84.3	NR	2.9 (max)	1
*Ae. koreicus*	42.9	NR	6.8 (max)	1
*An. albimanus*	17.8	3.35	0	1

## Data Availability

No new datasets were generated or analysed during the current study. All data discussed in this review were obtained from previously published studies cited in the manuscript.

## Abbreviations

The following abbreviations are used in this manuscript:

Abbreviation	Definition
*Ae.*	*Aedes*
*An.*	*Anopheles*
CHIKV	Chikungunya Virus
*Cs.*	*Culiseta*
*Cx.*	*Culex*
DR	Dissemination Rate
DRC	Democratic Republic of the Congo
ECSA	East/Central/South African Genotype
ECSA-IOL	East/Central/South African–Indian Ocean Lineage
EIP	Extrinsic Incubation Period
*Hg.*	*Haemagogus*
HICoE	Higher Institution Centre of Excellence
IFA	Immunofluorescence assay
IOL	Indian Ocean Lineage
IR	Infection Rate
MIR	Minimum Infection Rate
NA	Not Applicable
NGS	Next-Generation Sequencing
NR	Not Recorded
PCR	Polymerase Chain Reaction
*Ps.*	*Psorophora*
qPCR	Quantitative Polymerase Chain Reaction
RT-PCR	Reverse Transcription Polymerase Chain Reaction
RT-qPCR	Reverse Transcription Quantitative Polymerase Chain Reaction
TE	Transmission Efficiency
TIDREC	Tropical Infectious Diseases Research and Education Centre
TR	Transmission Rate
UM	Universiti Malaya
WHO	World Health Organization
*Wy.*	*Wyeomyia*
